# Engineering substrate promiscuity in 2,4-dichlorophenol hydroxylase by *in silico* design†

**DOI:** 10.1039/c8ra03229g

**Published:** 2018-06-08

**Authors:** Ye Wang, Chengkai Zhang, Song An, Xuexun Fang, Dahai Yu

**Affiliations:** College of Life Science, Jilin University 2699 Qianjin Street Changchun 130012 P. R. China; Key Laboratory for Molecular Enzymology and Engineering of Ministry of Education, College of Life Science, Jilin University 2699 Qianjin Street Changchun 130012 P. R. China yudahai@jlu.edu.cn +86-431-85155240 +86-431-85155249

## Abstract

2,4-Dichlorophenol hydroxylase (2,4-DCP hydroxylase) is a key enzyme in the degradation of 2,4-dichlorophenoxyacetic acid in the hydroxylation step in many bacteria. Our previous study demonstrated that a 2,4-DCP hydroxylase (TfdB-JLU) exhibits broad substrate specificity for chlorophenols (CPs) and their homologues. In this study, TfdB-JLU has been engineered by rational design to further broaden its substrate scope towards CPs. We dissect the architectures of enzymes from oxidoreductase families to discover their underlying structural sources of substrate promiscuity. A homology model of TfdB-JLU has been built and docking experiments of this homology model with its natural substrate 2,4-DCP reveal that the phenyl rings of 2,4-DCP form strong interactions with residues His47, Ile48, Trp222, Pro316, and Phe424. These residues are found to be important for substrate binding in the active site. Then, the site-directed mutagenesis strategy has been applied for redesigning substrate promiscuity in TfdB-JLU. The TfdB-JLU-P316Q variant obtained shows a significant enhancement of activity (up to 3.4-fold) toward 10 CP congeners compared to wild-type TfdB-JLU. Interestingly, the active improvements of TfdB-JLU-P316Q toward CP congeners show significant difference, especially for active improvements of positional congeners such as 3-CP (1.1-fold) compared to 4-CP (3.0-fold), as well as 2,3-DCP (1.2-fold) compared to 2,5-DCP (3.4-fold). Structural analysis results indicate that the improvement in substrate promiscuity of the variant enzyme compared to the wild-type enzyme is possibly due to the increase of non-bonding interaction. The results suggest that exploiting enzyme–substrate promiscuity is promising, which would provide a starting point for designing and engineering novel biological catalysts for pollution removal.

## Abstract 2

*In silico* designed 2,4-DCP hydroxylase exhibits broader substrate promiscuity for chlorophenols than that of the wild-type enzyme.

## Graphical abstract

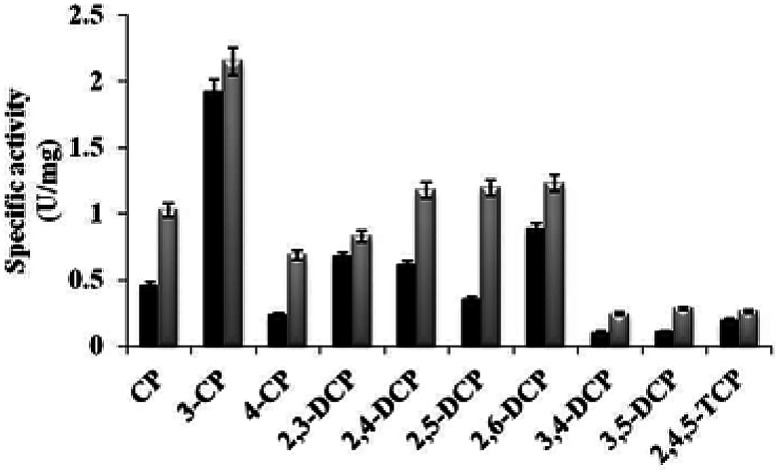

## Introduction

Enzymes are attractive catalysts because of their promiscuity and their ability to perform highly regio-, chemo- and stereoselective transformations. Enzymes that display promiscuous behavior can be classified into reaction conditions of promiscuity, substrate promiscuity, catalytic promiscuity and alternate-site promiscuity.^1^ Enzyme promiscuity is the engine of evolutionary innovation which can dramatically enhance the utility of biocatalysis in biotechnology. It has thus attracted significant attention from chemists and biochemists.^2^

Substrate promiscuity (also known as substrate ambiguity or broad substrate specificity) of enzyme refers to the catalysis of the same reaction with a range of substrates. Enzyme with substrate promiscuity displays several advantages since they can be used to transform substrates apart from the native substrates or chemocatalysis.^3^ Thus, substrate promiscuity provides useful starting points for exploring novel enzymes in engineered pathways for biodegradation of widely used chemicals pollutions.^4^

Numerous enzyme classes has been reported to exhibit substrate promiscuity including cytochrome P450s,^5,6^ kinase,^7,8^ phosphatases,^9^ acylaminoacyl peptidase,^10^ DNA methyltransferase,^11^ cyclic dipeptide prenyltransferase,^12^ glutathione S-transferases,^13^ laccases^14^ and lipases.^15^ Among these enzymes, 2,4-dichlorophenol (2,4-DCP) hydroxylase (EC 1.14.13.20) is the best candidate for developing a biocatalyst for chlorophenols (CPs) due to its high hydroxylation activities toward CPs compared to those of the reported cytochrome P-450s and laccases to the best of our knowledge.^16^ Our previous study demonstrates that a 2,4-DCP hydroxylase (TfdB-JLU) exhibits broad substrate specificity for CPs and their homologues.^17^ However, this enzyme exhibited high activities toward only limited CP congeners. Thus, there has been substantial interest in expanding the substrate scope of TfdB-JLU toward more CP congeners.

Protein engineering has emerged as a powerful method to improve or alter the properties of enzymes during the past two decades. Computer modelling, directed evolution, gene shuffling, site-directed mutagenesis, and rational design (or combinations thereof) have been successfully applied to engineer the substrate promiscuity of enzyme.^18,19^ For example, Wu *et al.* reported the evolution of *Candida antarctica* lipase B mutants with broad substrate scope toward α-chiral esters by directed evolution based on iterative saturation mutagenesis.^20^ Cassidy J engineered an alcohol dehydrogenase from the halophilic archaeon *Haloferax volcanii* by rational design to greatly broaden its substrate scope toward the conversion of a range of aromatic substrates.^21^ However, few studies have been performed to elucidate the substrate promiscuity mechanism of TfdBs to date.

This study mainly aims to discover underlying structural basis of substrate promiscuity of TfdB-JLU toward CPs and to engineer TfdB-JLU variants with higher activity toward CPs. The study is initialized by using SWISS-MODEL to do the homology model of TfdB-JLU. Molecular docking study has indicated that His47, Ile48, Trp222, Ppo316, and Phe424 might be the key amino acid for its substrate promiscuity. Then in order to improve the substrate promiscuity of the enzyme, active-site saturation test and site-directed mutagenesis strategy have been applied for redesigning substrate promiscuity of TfdB-JLU.

## Materials and methods

### Materials

CPs of analytical grade are purchased from J&K Scientific Ltd. (Shanghai, China). Other chemicals of analytical grade are obtained from Sigma. Recombinant *Escherichia coli* DH5α containing the TfdB-JLU gene for 2,4-DCP hydroxylase expression is from our lab.

### Methods

#### Sequence alignment and homology modeling of TfdB-JLU

The amino acid sequence of TfdB-JLU is collected from uncultured bacterium in the NCBI protein database (GenBank no. ACV31372.1). The initial amino acid sequence is analyzed using SWISS-MODEL (http://swissmodel.expasy.org/) to predict and select the template structure of the target models.^22–24^ 2-Hydroxybiphenyl 3-monooxygenase (PDB ID: 5brt) sharing highest sequence identity (41.90%) with TfdB-JLU is thus chosen as template protein for homology modeling.^25^ The automated sequence alignment of the 5brt and TfdB-JLU are carried out using the Molecular Evolutionary Genetics Analysis version 5 (MEGA5) program.^26^ The sequence alignment results are analyzed by Discovery Studio 4.5 Visualizer (DS 4.5) software (Accelrys, San Diego, CA). The structure of TfdB-JLU is generated using SWISS-MODEL server, and the output of model is a standard coordinate file in the PDB format. The quality of TfdB-JLU and 5brt models are validated by PROCHECK, Verify-3D Server (https://services.mbi.ucla.edu/SAVES/), ProSA web (https://prosa.services.came.sbg.ac.at/prosa.php). Molecular energy was minimized using the energy minimization module of DS 4.5.^27^

#### Molecular docking

Docking is frequently used to predict the binding orientation of small molecule candidates to their protein targets in order to in turn predict the affinity and activity of the small molecule. The Autodock 4.2 is used to estimate the potential substrate binding position for the enzyme as molecular docking program.^28^ The 3D structures of TfdB-JLU are built from homology modeling method. The variants of TfdB-JLU are edit by DS 4.5 software. The substrate structures of CP congeners are also generated by DS 4.5 software. To get a better statistics and clustering, each docking is performed twice, and the parameter value of maximum number of energy evaluations, initial population size and individual LGA executions is set to 25 000 000, 300 and 100, respectively.^29,30^ The predicted complexes are optimized and ranked according to a free-energy scoring function that is based on a linear regression analysis and the AMBER force field.^31^ The results of molecular docking are analyzed by DS 4.5 software.

#### Protein expression and purification

The recombinant *E. coli* is cultivated in LB medium containing 30 μg kanamycin per ml and 34 μg chloramphenicol per ml at 37 °C. Protein expression is cultivated at an OD600 of 0.6 and induced at 16 °C by the addition of 0.2 mM isopropyl-β-D-1-hiogalato-side (IPTG) (Fisher Scientific, Dingguo, BJ). After 15 h incubation, the cell pellets are harvested by centrifugation at 12 000 rpm and wash with 50 mM pH 7.5 sodium phosphate buffer for three times. For the preparation of crude extract, cells are suspended in 30–35 ml sodium phosphate buffer with 0.6 mM PMSF (phenylmethylsulfonyl fluoride) through ultrasonic breakage. Then, the lysate is centrifuged using a high speed freezing centrifuge (Fisher Scientific, Beckman Coulter, USA) at 15 000 rpm for 20 min at 4 °C. The supernatant is transferred onto a Hislink™ column rinsed with wash buffer (50 mM sodium phosphate buffer with 0.6 mM PMSF, 10 mM imidazole, pH 7.5). The protein supernatant is loaded onto a nickel–nitrilotriacetic agarose resin (Qiagen, Germany) equilibrated with the same buffer. After washing with 5 column volumes of the wash buffer (40 mM imidazole), the bound enzyme is eluted with the elution buffer (200 mM imidazole). Then the fractions are concentrated by ultrafiltration and then are diafiltered with 50 mM sodium phosphate buffer, pH 7.5, containing 10% (v/v) glycerol. Samples are stored at −80 °C for further analysis.

#### Enzyme assay and characterization

The enzyme assay is determined as described previously.^32^ One unit of activity is defined as the amount of enzyme required to consume 1 μmol substrate per min at 25 °C. Protein concentrations are determined by the BCA method (Novagen® BCA Protein Assay Kit) using bovine serum albumin as the standard. A difference with a *p* < 0.05 is considered significant. Results are shown as mean ± SEM of *n* indicated in each case.

## Results and discussion

### Sequence alignment, homology modeling of TfdB-JLU and model evaluation

In order to engineer the promiscuity of the enzyme toward the CPs by rational protein design, it is mandatory to understand the mechanism of this reaction and to have a reliable structural model of this enzyme. In this study, an *in silico* approach is used to obtain the three-dimensional structure of TfdB-JLU. While the TfdB has been investigated in many studies, the crystal structure of this enzyme has not been solved yet. Thus, it is essential to find a homologous enzyme, which has been characterized in detail. Templates with high protein sequence identity are obtained by SWISS-MODEL server (Table S.1†). Then we chose 2-hydroxybiphenyl-3-monooxygenase (PDB ID: 5brt_A) which has been successfully crystallized at 2.30 Å by Fishman *et al.* as our template due to its highest homology (41.9%) and its possession of binding site of both the substrate and FAD.^25^ Fig. S.1† shows the sequence alignment between TfdB-JLU and 5brt protein. Then TfdB-JLU model structure is made by MEGA5. Fig. S.2† shows the 3D structure alignment between the TfdB-JLU model and the 5brt with their root mean square deviation (RMSD) value of 1.85 Å. FAD is added during the docking study since 5brt structural information suggested that TfdB-JLU contains the FAD binding domain as shown in Fig. S.3.† NADPH, however, is not added in TfdB-JLU modeling, since NADPH binding domain requires strong positively charged environment, which is lacked in TfdB-JLU.^33,34^

**Fig. 1:**
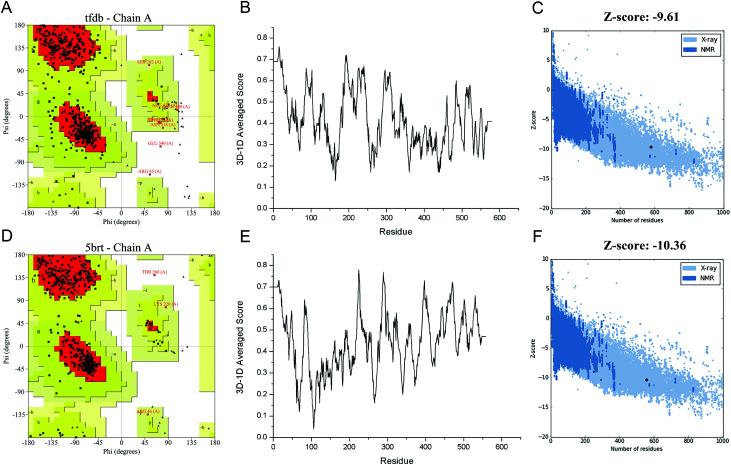
Structure validation of target (TfdB-JLU) model and template (5brt) model. (A and D) Ramachandran plot of TfdB-JLU and 5brt, respectively. (B and E) 3D-1D averaged score plot of TfdB-JLU and 5brt, respectively. (C and F) *Z*-score plot of TfdB-JLU and 5brt, respectively.

Protein structure evaluation is then assessed by online server as described above. The stereochemical quality of the model is acceptable as shown in the Ramachandran plot obtained by PROCHECK.^35^ The amino acid residues in the TfdB-JLU and 5brt structure are 85.0% and 89.6% in most favored regions, 13.4% and 9.7% in additional allowed regions, 1.2% and 0.4% in generously allowed regions, and only 0.4% and 0.2% in disallowed regions, respectively (Table S.2† and Fig. 1A and D). To analyze the compatibility of an atomic model (3D) with its own amino acid sequence (1D) by assigning a structural class based on its location and environment (alpha, beta, loop, polar, nonpolar *etc.*) and comparing the results to good structures, the TfdB-JLU model is evaluated with Verify 3D.^36^ 98.1% and 96.1% of residues had an averaged 3D-1D score ≥ 0.2 in the TfdB-JLU and 5brt model (Fig. 1B and E). This suggests that atomic model (3D) is compatible with its amino acid sequence (1D) and the structure is identified as stable conformation. Finally the TfdB-JLU model is analyzed using ProSA-web server. The *Z*-score is used to indicate overall model quality.^37^ The *Z*-score values are calculated by the highlighted black dot displayed in a plot (Fig. 1C and F). Similarity between −9.61 *Z*-score value of TfdB-JLU model and −10.36 *Z*-score of the 5brt model suggests high model quality. The above results show that the predicted structures conformed well to the stereochemistry, which indicates reasonably good quality.

### Key amino acid prediction affecting substrate promiscuity by molecular docking

**Fig. 2:**
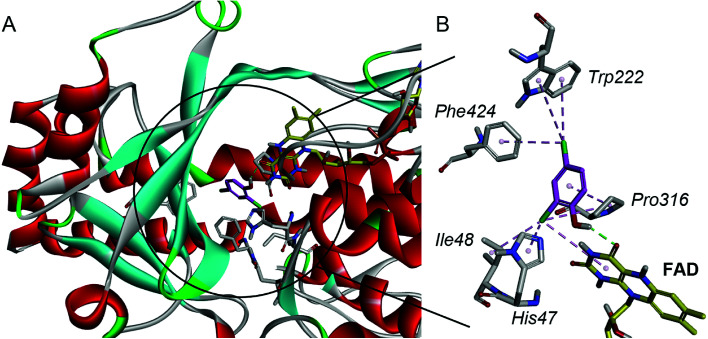
The interaction schematic diagram of 2,4-DCP with the active site of TfdB-JLU model. (A) 2,4-DCP (magenta) is located in the hydrophobic pocket of the active site of TfdB-JLU (gray sticks) and forms a hydrogen bond (green dashed lines) with FAD (yellow sticks). (B) The schematic diagram of the detail non-bond interaction formations of 2,4-DCP (magenta) with the active site of TfdB-JLU. It shows that 2,4-DCP forms one hydrogen bond (green dashed lines) with carbonyl oxygen atom of the FAD (yellow sticks) isoalloxazine ring, and eight non-bond interaction (pink dashed lines) with amino acid residues His47, Ile48, Trp222, Pro316 and Phe424 of TfdB-JLU (gray sticks).

For a better understanding of the substrate and enzyme interaction mechanism, molecular docking experiment is conducted with the modeling TfdB-JLU and 2,4-DCP. Docking results shows 2,4-DCP is located in the hydrophobic pocket of the active site, and forms hydrogen bond interaction with carbonyl oxygen atom of the isoalloxazine ring of FAD (as shown in Fig. 2A). Tables 1 and 2 gives the detail non-bond parameters of 2,4-DCP with TfdB-JLU. As Fig. 2B shows, residues His47, Ile48, Trp222, Pro316 and Phe424 of TfdB-JLU are believed to play key role in substrate binding, since they form hydropholic and halogen interactions with 2,4-DCP. These amino acid residues are thus used for further study.

**Table 1:** Hydrogen bond parameters of 2,4-DCP and TfdB-JLU_WT/TfdB-JLU_P316Q

Receptor	Donors Atom	Receptor Atom	Distances (Å)^a^
TfdB-JLU_WT	2,4-DCP: H	TfdB-JLU_WT: FAD: O4	2.21
TfdB-JLU_P316Q	TfdB-JLU_P316Q: His47: HE2	2,4-DCP: O	1.81
	TfdB-JLU_P316Q: FAD: H3	2,4-DCP: O	2.52

a The length of the hydrogen bonds.

**Table 2:** Hydropholic and halogen interaction parameters of 2,4-DCP with TfdB-JLU_WT

Types	Form chemistry	To chemistry	Distances (Å)
Alkyl	2,4-DCP: Cl1	TfdB-JLU_WT: Ile48	4.14
Alkyl	2,4-DCP: Cl1	TfdB-JLU_WT: Pro316	5.07
Pi-alkyl	TfdB-JLU_WT: His47	2,4-DCP: Cl1	3.91
Pi-alkyl	TfdB-JLU_WT: Trp222	2,4-DCP: Cl12	5.12
Pi-alkyl	TfdB-JLU_WT: Trp222	2,4-DCP: Cl12	4.40
Pi-alkyl	TfdB-JLU_WT: Phe424	2,4-DCP: Cl12	4.60
Pi-alkyl	FAD	2,4-DCP: Cl1	5.43
Pi-alkyl	2,4-DCP	TfdB-JLU_WT: Pro316	4.07

The amino acid residues His47, Ile48, Trp222, Pro316 and Phe424 of TfdB-JLU are individually mutated to alanine using DS 4.5 software. 2,4-DCP is then docked into theses protein variants, respectively. The free energy of binding of 2,4-DCP to TfdB-JLU variants increased in comparison with that of 2,4-DCP to TfdB-JLU (Table 3). The variants exhibiting the greatest increase in free energy of binding with substrate are TfdB-JLU_H47A (−4.70 kcal mol^−1^), TfdB-JLU_P316A (−4.80 kcal mol^−1^) and TfdB-JLU_W222A (−4.87 kcal mol^−1^). His47 in the active site is suggested to play the key role in substrate deprotonation,^38^ and this residue should not be mutated. Due to the above reasons, Pro316 and Trp222 are selected as key residues affecting substrate promiscuity since the mutation of these two sites to alanine results the increase of free energy, and do not change the binding site significantly.

**Table 3:** Estimated free energy of binding of 2,4-DCP and TfdB-JLU_WT/TfdB-JLU-mutants

Proteins	Energy (kcal mol^−1^)
TfdB-JLU_WT	−5.14
TfdB-JLU_H47A	−4.70
TfdB-JLU_I48A	−5.00
TfdB-JLU_W222A	−4.87
TfdB-JLU_P316A	−4.80
TfdB-JLU_F424A	−4.98

**Fig. 3:**
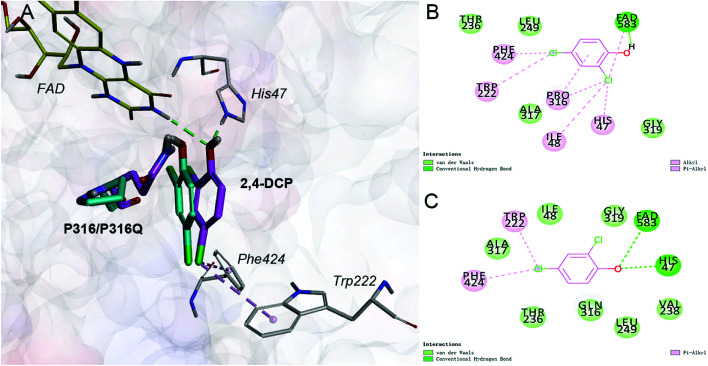
(A) Comparison of the interaction of 2,4-DCP-TfdB-JLU_P316Q complex with 2,4-DCP-TfdB-JLU complex. The ligand 2,4-DCP and amino acid residue P316Q in 2,4-DCP-TfdB-JLU_P316Q complex is shown by magenta stick, the ligand 2,4-DCP and amino acid residue P316 in 2,4-DCP-TfdB-JLU complex is shown by cyan stick, FAD is shown by yellow line, and the amino acid residues in TfdB-JLU_P316Q are shown by white line. (B) The schematic diagram of the detail non-bond interaction formations of 2,4-DCP with the active site of TfdB-JLU_WT. (C) The schematic diagram of the detail non-bond interaction formations of 2,4-DCP with the active site of TfdB-JLU_P316Q. The hydrogen bond interactions are shown by green dashed line, and the hydrophobic interactions are shown by pink dashed lines.

Saturation mutagenesis is employed in protein engineering and genome-editing efforts to generate libraries that span amino acid design space for functional improvements. In this study, we perform site-saturation mutations for Pro316 and Trp222. 2,4-DCP is docked into these protein variants, respectively. As the Table S.3† shows, the free energy is reduced when substrate is docked into TfdB-JLU_P316Q. Hence, this mutation has a catalytic effect on binding. In terms of TfdB-JLU_P316Q, although the non-bond interaction of substrate and TfdB-JLU_P316Q is reduced (Table S.4†), an increase in hydrogen bonding is observed (Table 1). As shown in Fig. 3, the mutation of P316Q increases steric hindrance at the active site of enzyme and pushes the substrate to the direction of H47. This mutation results in the formation of hydrogen bonding between the substrate and H47, which might be the reason for the significant decrease in the binding free energy of the substrate and the TfdB-JLU_P316Q. Docking experiments have also been performed on P316N (least change in free energy of binding) and P316I (highest increase in free energy of binding). As shown in Fig. S.5†, little bonding change has been observed in cases of TfdB-JLU_WT and TfdB-JLU_P316N. However, the interaction of substrate with TfdB-JLU-P316I differed greatly from that of TfdB-JLU_WT with the disappearance of hydrogen bond. No corresponding saturated mutants for Trp222 show reduced free energy value (Table S.3†). Thus, we select TfdB-JLU_P316Q for further experiments.

### Substrate promiscuity and activity of TfdB-JLU_P316Q toward 10 CP congeners

**Fig. 4:**
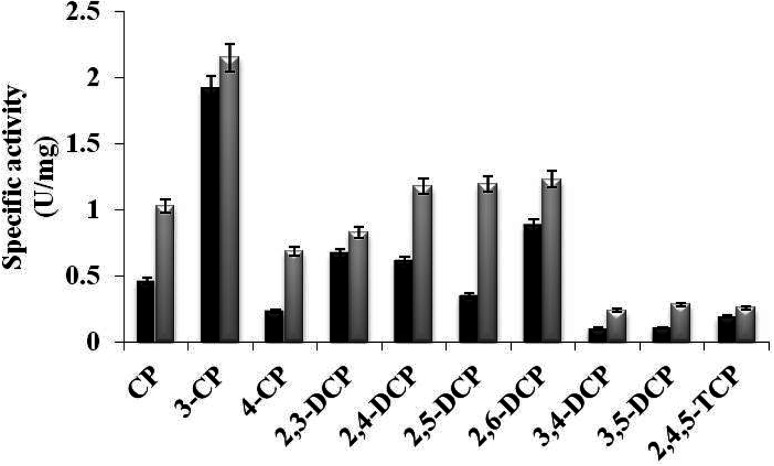
Specific activity of TfdB-JLU_WT (black) and TfdB-JLU_P316Q (gray) toward CPs. Abbreviations: CP, chlorophenol; DCP, dichlorophenol; TCP, trichlorophenol.

In the present study, TfdB-JLU_P316Q activities toward 10 selected CP congeners are investigated at 25 °C. As summarized in Fig. 4, TfdB-JLU_P316Q exhibits high activities toward all the 10 CP congeners compared to those of TfdB-JLU. This result supports our modelling study that P316 is the key residue for enzyme functional engineering. The results in Fig. 4 shows that TfdB-JLU exhibits low activities toward 3,4-DCP, 3,5-DCP, whereas the activities of TfdB-JLU_P316Q toward 3,4-DCP and 3,5-DCP improve by 2.36-fold and 2.66-fold, respectively. This result suggests that TfdB-JLU_P316Q exhibits substrate promiscuity toward selected CP congeners and its substrate scope is thus broader than that of TfdB-JLU because of its high activity. Enzymatic activity improvement patterns observed for the mono-, di- and tri-CPs are quite different. Interestingly, enzymatic activity increases by 3.45, 2.98, 2.65, 2.36 and 2.23-fold for some CP congeners including 2,5-DCP, 4-CP, 3,5-DCP, 3,4-DCP and CP, respectively. The activity of TfdB-JLU_P316Q for natural substrate 2,4-DCP increases only by 1.91-fold compared to that of TfdB-JLU.^11^ The activity improvements of TfdB-JLU_P316Q for 3-CP, 2,3-DCP, 2,6-DCP, and 2,4,5-TCP are only found to be 1.12, 1.23, 1.41 and 1.36-fold higher than that of TfdB-JLU, respectively. Moreover, the enzyme activity improvement toward CP congeners is found related to the chlorine ring substitution patterns of specific CP congeners (Fig. 4). The enzyme activity of TfdB-JLU_P316Q for 3-CP are approximately 3-fold higher than that for 4-CP. However, in terms of activity improvement, the activity improvement of TfdB-JLU_P316Q for 3-CP is about 2.6-fold lower than that for 4-CP. Similar results are also observed for those of 2,5-DCP and 2,6-DCP. The above results suggest that P316 residue is a key residue for exploring substrate promiscuity of TfdB-JLU toward CPs, especially for those of unfavourable CPs. The differences between these enzymatic activities are statistically significant (*p* < 0.05).

### TfdB-JLU substrate promiscuity mechanism prediction

In this study, we would like to propose a preliminary assumption on the substrate promiscuity mechanism of TfdB-JLU toward CPs based on the structure and activity analyses. Molecular docking is used to analyze the interaction between the CPs ligands and TfdB-JLU_P316Q. An overall correlation of variation tendency is observed between the calculated free energy of binding and the substrate promiscuity. As Table S.5† shows, all ten CP congeners have the lower binding free energy with TfdB-JLU_P316Q than those with TfdB-JLU. The result in Fig. S.4† shows that the non-bond interactions between ligands such as 4-CP, 2,5-DCP and 3,5-DCP and TfdB-JLU_P316Q are stronger than those of ligands with TfdB-JLU_WT. Tables S.6–S.12† gives the detail parameters for these non-bonding interactions. Although a correlation is not observed between the calculated free energy of binding and the specific activity. Binding free energies of TfdB-JLU_P316Q with all CP congeners are lower than those of wild-type enzyme, whereas the specific activities of TfdB-JLU_P316Q toward all CP congeners are higher than those of TfdB-JLU_WT (Fig. S.6†). Thus, the higher decrease in free energy of binding might not necessarily lead to subsequent increased substrate promiscuity. It is notable that the higher decrease in free energy of binding dose not leads to subsequent increase in enzyme activity as shown in Fig. S.6.† For example, 2,4,5-TCP is shown to have the lowest free energy of binding out of the ten congeners (Table S.5†); but the specific activities of both TfdB-JLU_WT and TfdB-JLU_P316Q are the lowest for 2,4,5-TCP out of all the ten congeners (Fig. 4). In addition, the increase of free energy with the most extent does not witness the decrease of the specific activity with the most extent. For example, when 3,5-DCP is used as substrate, as the free energy slightly drops 0.03 kcal mol^−1^ (from −5.19 kcal mol^−1^ for TfdB-JLU_WT to −5.22 kcal mol^−1^ for TfdB-JLU_P316Q), the specific activity increases in 2.66-fold (Table S.5†). Comparatively, when 3-DCP is used as substrate, the free energy drops 0.20 kcal mol^−1^ (from −4.55 kcal mol^−1^ for TfdB-JLU_WT to −4.75 kcal mol^−1^ for TfdB-JLU_P316Q) (Table S.5†), but the increase in specific activity is only 1.12-fold. Therefore, the binding of the ligands to TfdB-JLU_P316Q is better than the binding to TfdB-JLU_WT due to not only its low binding free energy, but also the increase of hydrogen bond interaction, hydrophobic and halogen interactions. This result well supports the higher substrate promiscuity of TfdB-JLU_P316Q compared to TfdB-JLU_WT. And this engineered enzyme would be a good candidate for providing a starting point for designing and engineering novel biological catalyst for all CP congeners' removal.

## Conclusions

This study exemplifies a site-directed mutagenesis of a 2,4-DCP hydroxylase TfdB-JLU based on rational design, and evolutionized variants exhibit broad substrate promiscuity for CPs than the wild-type TfdB-JLU. The modeling structure of TfdB-JLU is successfully used to interpret the results of *in vitro* evolution experiments and engineer substrate promiscuity. P316 is found to be a key amino acid associated with substrate promiscuity through saturation mutation screening and enzyme assay validation. The study of structures of TfdB-JLU and site-specific variants sheds light on the enzyme substrate promiscuity mechanism and will further aid protein engineering of biocatalysts with potential industrial applications.

## Conflicts of interest

There are no conflicts to declare.

## Supplementary Material

RA-008-C8RA03229G-s001
