# Supplementary material for: Engineering substrate promiscuity in 2,4-dichlorophenol hydroxylase by in silico design
Source: RSC Adv. 2018 Jun 8;8(38):21184–90. doi: 10.1039/c8ra03229g (PMC9080915; doi:10.1039/c8ra03229g)
Supplement: RA-008-C8RA03229G-s001 [file RA-008-C8RA03229G-s001.pdf]

Electronic Supplementary Material (ESI) for RSC Advances.
This journal is © The Royal Society of Chemistry 2018

Supplementary Figures

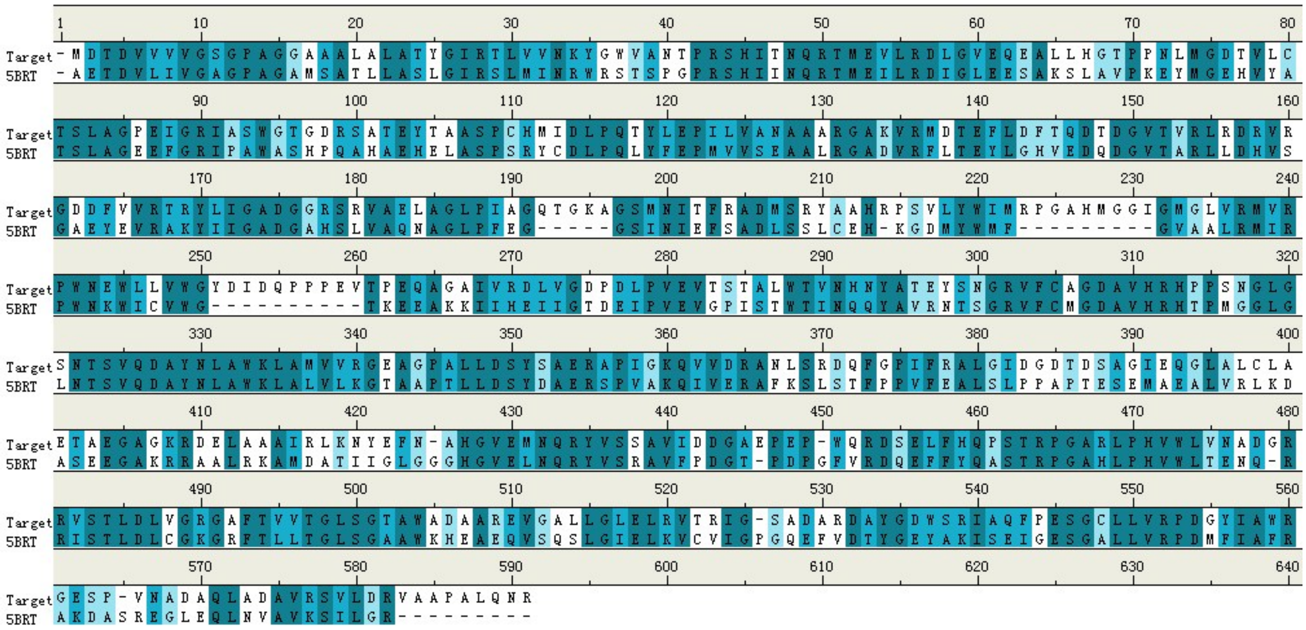

Fig S.1 Sequence alignment between target protein TfdB-JLU and template protein 2-hydroxybiphenyl-3-monooxygenase. Sequence identity is 41.90%.

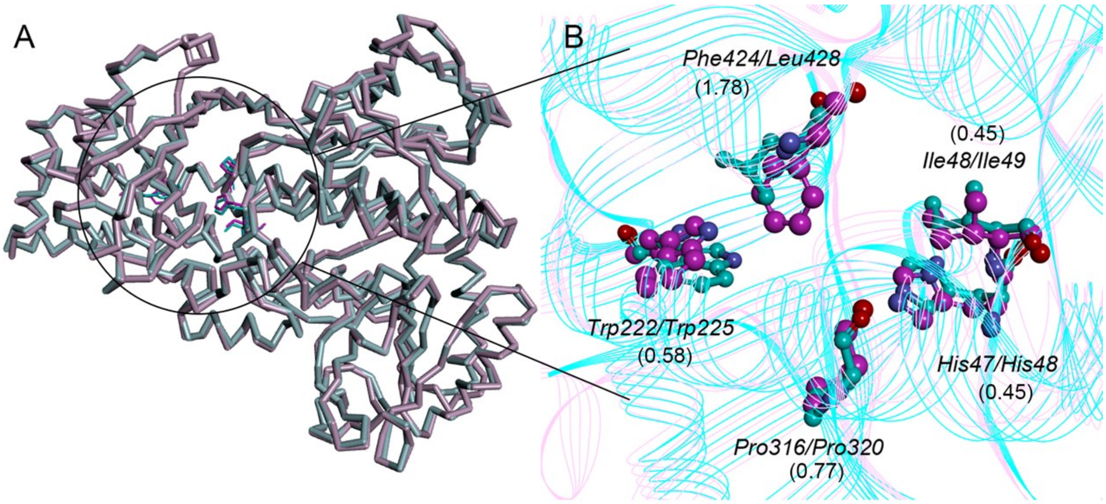

Fig S.2 **A** stereo view of the Cα backbone atoms of the homology model of TfdB (violet) superimposed with 5brt (cyan). Their root mean square deviation (RMSD) value was 1.85 Å. **B** The schematic diagram shows the detail of key residues: His47, Ile48, Trp222, Pro316, Phe424 of TfdB-JLU (magenta ball and stick) and His48, Ile49, Trp225, Pro320, Leu428 of 5brt (light cyan ball and stick). The values in parentheses stand for the RMSD value of key residues of TfdB-JLU versus matching residues of 5brt.

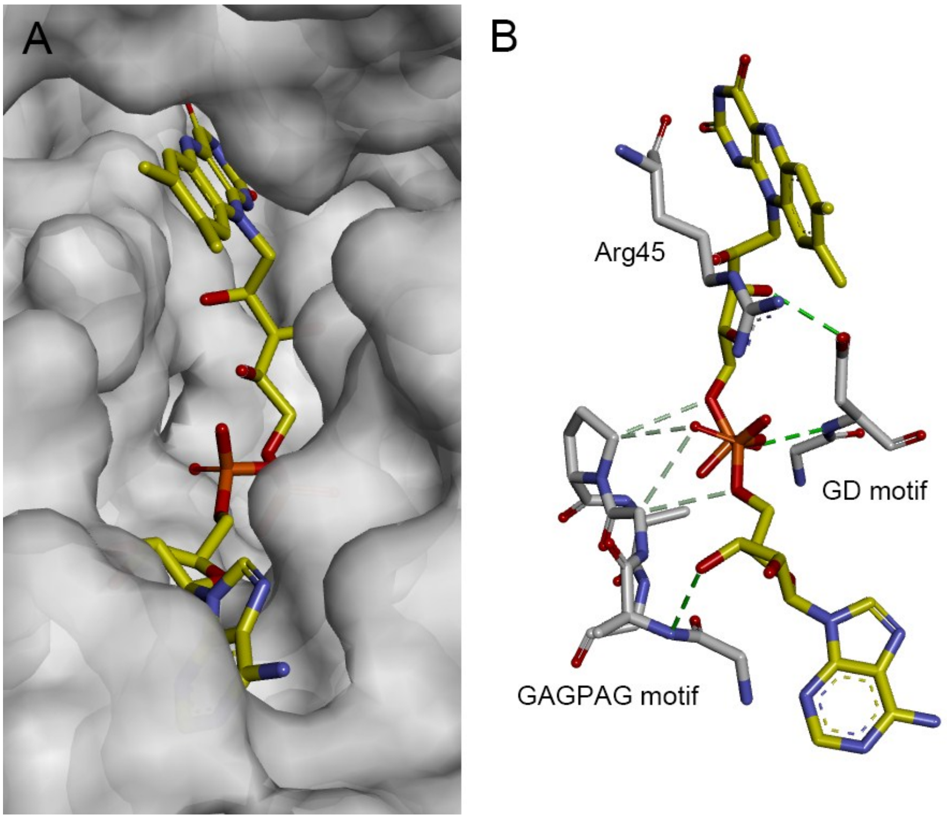

Fig S.3 FAD binding site of TfdB-JLU. **A**. Surface presentation of the FAD binding cavity. **B**. The extensive hydrogen bond interactions of FAD in the binding site. The hydrogen bonds are shown as green dashed lines.

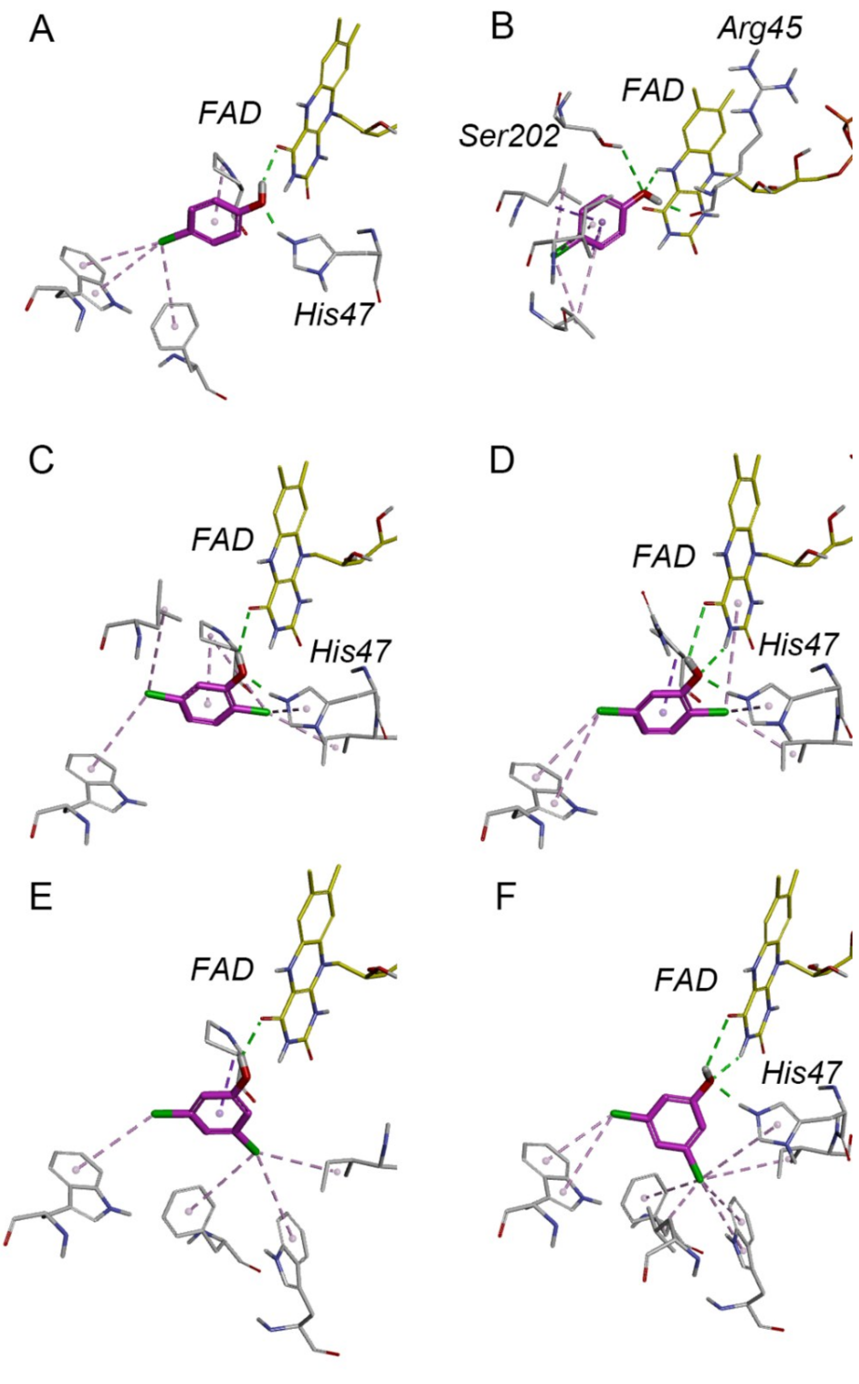

Fig. S.4 The interaction of certain ligands with the active site of TfdB-JLU_WT (A, C, E)/ TfdB-JLU_P316Q (B, D, F). The ligands are 4-CP (in A, B), 2,5-DCP (in C, D) and 3,5-DCP (in E, F), respectively. The ligands are shown by magenta stick, FAD is shown by yellow line, and the residues in TfdB-JLU_WT/TfdB-JLU_P316Q are shown by gray line. The hydrogen bond interactions are shown by green dashed line, and the hydrophobic and halogen interactions are shown by pink dashed lines. The residues and FAD labeled have the hydrogen bond interaction with ligands.

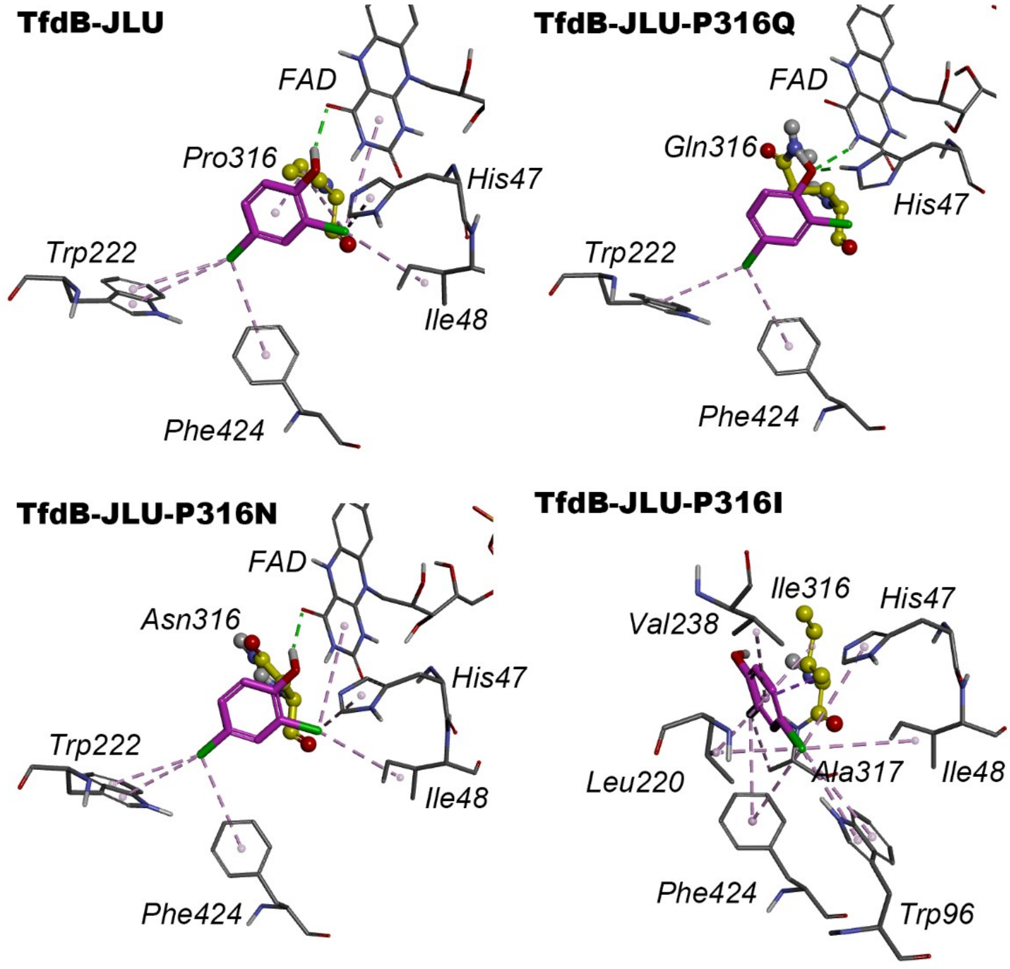

Fig. S.5 The schematic diagram of the detail non-bond interaction formations of 2,4-DCP with the active site of TfdB-JLU_WT, TfdB-JLU-P316Q, TfdB-JLU-P316N and TfdB-JLU-P316I. The 2,4-DCP is shown by magenta stick, the 316 residue of TfdB-JLU_WT/TfdB-JLU-mutants is shown by yellow ball and stick, the residues of TfdB-JLU_WT/TfdB-JLU-mutants and FAD are shown by gray line. The hydrogen bond interactions are shown by green dashed line, and the hydrophobic and halogen interactions are shown by pink dashed lines.

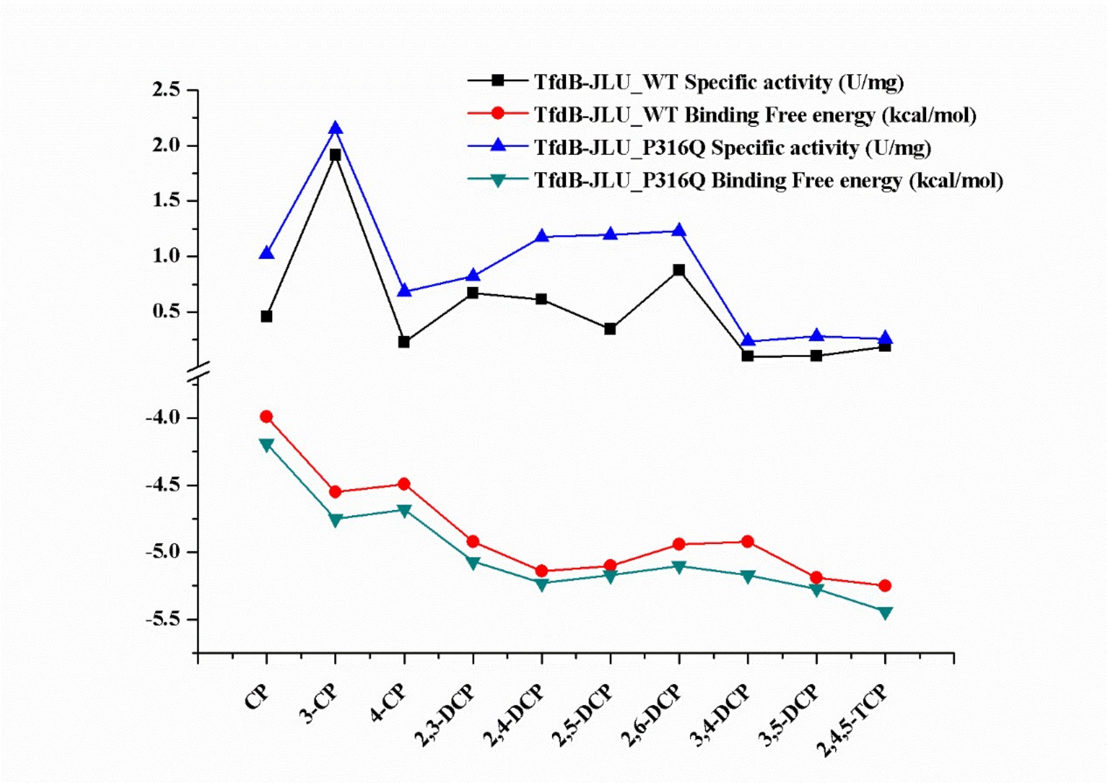

Fig. S.6 Free energies of binding of CPs with TfdB-JLU_WT and TfdB-JLU_P316Q, and specific activities of TfdB-JLU_WT and TfdB-JLU_P316Q toward CPs.

Supplementary Tables

Table S.1 Template search using SWISS-MODEL server.

Template		Identity	Coverage		Name	Ligands	Method	Oligo state
PDB ID	Chain	(%)	Range	Percent				
4cy6	A	41.90	1-582	0.98	2-hydroxybiphenyl-3-monooxygenase	None	X-ray 2.76Å	homo-tetramer
5brt	A	41.90	1-582	0.98	2-hydroxybiphenyl-3-monooxygenase	FAD, CH9	X-ray 2.30Å	homo-tetramer
3ihg	A	37.28	1-582	0.88	RdmE	FAD, VAK, SO4	X-ray 2.49Å	monomer
3ept	A	32.94	4-563	0.85	RebC	FAD, NA	X-ray 2.97Å	monomer

Table S.2 Ramachandran Plot statistics for the target (TfdB-JLU) and template (5brt) models.

Ramachandran Plot Statistics	TfdB-JLU		5brt	
	Nos.	%	Nos.	%
Residues in the most favoured regions	419	85.0	423	89.6
Residues in additional allowed regions	72	13.4	46	9.7
Residues in generously allowed regions	6	1.2	2	0.4
Residues in disallowed regions	2	0.4	1	0.2
Number of non-glycine and non-proline residues	499		472	
Number of end residues (excl. Gly and Pro)	3		8	
Number of glycine residues (shown as triangles)	52		49	
Number of proline residues	26		29	
Total number of residues	580	100	558	100

Table S.3 Estimated free energy of binding of 2,4-DCP and TfdB-JLU_P316 and TfdB-JLU_W222 saturated mutants.

Proteins	Energy (kcal/mol)	Proteins	Energy (kcal/mol)
TfdB-JLU	-5.14	TfdB-JLU	-5.14
TfdB-JLU_P316A	-4.80	TfdB-JLU_W222A	-4.88
TfdB-JLU_P316C	-5.00	TfdB-JLU_W222C	-4.88
TfdB-JLU_P316D	-5.13	TfdB-JLU_W222D	-4.87
TfdB-JLU_P316E	-5.04	TfdB-JLU_W222E	-5.11
TfdB-JLU_P316F	-4.64	TfdB-JLU_W222F	-4.86
TfdB-JLU_P316G	-4.77	TfdB-JLU_W222G	-4.97
TfdB-JLU_P316H	-4.63	TfdB-JLU_W222H	-4.90
TfdB-JLU_P316I	-4.51	TfdB-JLU_W222I	-4.96
TfdB-JLU_P316K	-4.66	TfdB-JLU_W222K	-4.94
TfdB-JLU_P316L	-4.64	TfdB-JLU_W222L	-4.94
TfdB-JLU_P316M	-4.73	TfdB-JLU_W222M	-4.88
TfdB-JLU_P316N	-5.13	TfdB-JLU_W222N	-4.88
TfdB-JLU_P316Q	-5.23	TfdB-JLU_W222P	-4.88
TfdB-JLU_P316R	-5.00	TfdB-JLU_W222Q	-4.85
TfdB-JLU_P316S	-4.67	TfdB-JLU_W222R	-4.88
TfdB-JLU_P316T	-4.57	TfdB-JLU_W222S	-4.87
TfdB-JLU_P316V	-4.72	TfdB-JLU_W222T	-4.88
TfdB-JLU_P316W	-4.81	TfdB-JLU_W222V	-5.08
TfdB-JLU_P316Y	-4.72	TfdB-JLU_W222Y	-4.88

Table S.4 Halogen interaction parameters of 2,4-DCP with TfdB-JLU_P316Q.

Types	Form chemistry	To chemistry	Distances(Å)*
Pi-Alkyl	TfdB_P316Q: Trp222	2,4-DCP: Cl12	4.95
Pi-Alkyl	TfdB_P316Q: Phe424	2,4-DCP: Cl12	4.88

*The length of the hydrogen bonds.

Table S.5 Estimated free energy of binding of ligands and TfdB-JLU_WT/TfdB-JLU_P316Q.

Ligands	With TfdB-JLU_WT Energy (kcal/mol)	With TfdB-JLU_P316Q Energy (kcal/mol)
CP	-3.99	-4.19
3-CP	-4.55	-4.75
4-CP	-4.49	-4.68
2,3-DCP	-4.92	-5.07
2,4-DCP	-5.14	-5.23
2,5-DCP	-5.10	-5.17
2,6-DCP	-4.94	-5.10
3,4-DCP	-4.92	-5.17
3,5-DCP	-5.19	-5.22
2,4,5-TCP	-5.25	-5.44

Table S.6 Hydrogen Bond parameters of ligands and TfdB-JLU_WT/TfdB-JLU_P316Q.

Substrates	TfdB-JLU_WT			TfdB-JLU_P316Q		
	Donors Atom	Receptor Atom	Distances(Å)*	Donors Atom	Receptor Atom	Distances(Å)*
4-CP	His47:HE2	4-CP: O	1.78	Ser202: HG	4-CP: O	2.96
	4-CP: H	FAD: O4	2.10	FAD: H5	4-CP: O	2.40
				4-CP: H	Arg45: O	1.98
2,5-DCP	His47: HE2	2,5-DCP: O	1.84	His47: HE2	2,5-DCP: O	1.74
	2,5-DCP: H	FAD: O4	2.50	FAD: H3	2,5-DCP: O	2.19
				2,5-DCP: H	FAD: O4	2.89
3,5-DCP	3,5-DCP: H	FAD: O4	2.12	His47: HE2	3,5-DCP: O	1.98
				FAD: H3	3,5-DCP: O	2.11
				3,5-DCP: H	FAD: O4	2.90

*The length of the hydrogen bonds.

Table S.7 Hydropholic and halogen interaction parameters of 4-CP with TfdB-JLU_WT.

Types	Form chemistry	To chemistry	Distances(Å)
Pi-Alkyl	TfdB-JLU_WT: Trp222	4-CP: Cl12	5.47
Pi-Alkyl	TfdB-JLU_WT: Trp222	4-CP: Cl12	4.45
Pi-Alkyl	TfdB-JLU_WT: Phe424	4-CP: Cl12	4.60
Pi-Alkyl	4-CP	TfdB-JLU_WT: Pro316	4.07

Table S.8 Hydropholic and halogen interaction parameters of 4-CP with TfdB-JLU_P316Q.

Types	Form chemistry	To chemistry	Distances(Å)
Pi-Sigma	TfdB-JLU_P316Q: Ile247:CG2	4-CP	3.90
Pi-Sigma	TfdB-JLU_P316Q: Leu249: CB	4-CP	3.70
Alkyl	4-CP : Cl12	TfdB-JLU_P316Q: Val238	3.70
Alkyl	4-CP: Cl12	TfdB-JLU_P316Q: Ile247	4.33
Alkyl	4-CP: Cl12	TfdB-JLU_P316Q: Leu249	5.01
Pi-Alkyl	4-CP	TfdB-JLU_P316Q: Val238	5.45

Table S.9 Hydropholic and halogen interaction parameters of 2,5-DCP with TfdB-JLU_WT.

Types	Form chemistry	To chemistry	Distances(Å)
Alkyl	2,5-DCP: Cl7	TfdB-JLU_WT: Leu249	5.15
Alkyl	2,5-DCP: Cl1	TfdB-JLU_WT: Ile48	4.03
Alkyl	2,5-DCP: Cl1	TfdB-JLU_WT: Pro316	5.24
Pi-Alkyl	TfdB-JLU_WT: His47	2,5-DCP: Cl1	3.93
Pi-Alkyl	TfdB-JLU_WT: Trp222	2,5-DCP: Cl7	4.90
Pi-Alkyl	2,5-DCP	TfdB-JLU_WT: Pro316	4.11

Table S.10 Hydropholic and halogen interaction parameters of 2,5-DCP with TfdB-JLU_P316Q.

Types	Form chemistry	To chemistry	Distances(Å)
Pi-Sigma	TfdB-JLU_P316Q: Gln316: CB	2,5-DCP	3.63
Alkyl	2,5-DCP: Cl1	TfdB-JLU_P316Q: Ile48	3.96
Pi-Alkyl	TfdB-JLU_P316Q: His47	2,5-DCP: Cl1	3.94
Pi-Alkyl	TfdB-JLU_P316Q: Trp222	2,5-DCP: Cl7	5.18
Pi-Alkyl	TfdB-JLU_P316Q: Trp222	2,5-DCP: Cl7	4.47
Pi-Alkyl	TfdB-JLU_P316Q: FAD	2,5-DCP: Cl1	5.39

Table S.11 Hydropholic and halogen interaction parameters of 3,5-DCP with TfdB-JLU_WT.

Types	Form chemistry	To chemistry	Distances(Å)
Pi-Sigma	TfdB-JLU_WT: Pro316: CB	3,5-DCP	3.41
Alkyl	3,5-DCP: Cl7	TfdB-JLU_WT: Ile48	4.12
Pi-Alkyl	TfdB-JLU_WT: Trp96	3,5-DCP: Cl7	4.92
Pi-Alkyl	TfdB-JLU_WT: Trp222	3,5-DCP: Cl13	4.69
Pi-Alkyl	TfdB-JLU_WT: Phe424	3,5-DCP: Cl7	4.66

Table S.12 Halogen interaction parameters of 3,5-DCP with TfdB-JLU_P316Q.

Types	Form chemistry	To chemistry	Distances(Å)
Alkyl	3,5-DCP: Cl7	TfdB-JLU_P316Q: Ile48	5.28
Alkyl	3,5-DCP: Cl7	TfdB-JLU_P316Q: Leu220	4.00
Pi-Alkyl	TfdB-JLU_P316Q: His47	3,5-DCP: Cl7	5.05
Pi-Alkyl	TfdB-JLU_P316Q: Trp96	3,5-DCP: Cl7	4.99
Pi-Alkyl	TfdB-JLU_P316Q: Trp96	3,5-DCP: Cl7	5.13
Pi-Alkyl	TfdB-JLU_P316Q: Trp222	3,5-DCP: Cl13	4.91
Pi-Alkyl	TfdB-JLU_P316Q: Trp222	3,5-DCP: Cl13	4.17
Pi-Alkyl	TfdB-JLU_P316Q: Phe424	3,5-DCP: Cl7	4.26
